# Fear of Foreigners: HIV-related restrictions on entry, stay, and residence

**DOI:** 10.1186/1758-2652-11-8

**Published:** 2008-12-16

**Authors:** Joseph J Amon, Katherine Wiltenburg Todrys

**Affiliations:** 1 HIV/AIDS Program, Human Rights Watch, New York, USA; 2 Independent Consultant, London, UK

## Abstract

Among the earliest and the most enduring responses to the HIV/AIDS epidemic has been the imposition by governments of entry, stay, and residence restrictions for non-nationals living with HIV and AIDS. Sixty-six of the 186 countries in the world for which data are available currently have some form of restriction in place. Although international human rights law allows for discrimination in the face of public health considerations, such discrimination must be the least intrusive measure required to effectively address the public health concern. HIV-related travel restrictions, by contrast, not only do not protect public health, but result in deleterious effects both at the societal level – negatively impacting HIV prevention and treatment efforts – and at the individual level, affecting, in particular, labor migrants, refugee candidates, students, and short-term travelers. Governments should repeal these laws and policies, and instead devote legislative attention and national resources to comprehensive HIV prevention, care, and treatment programmes serving citizens and non-citizens alike.

## Background

Governments often respond to emerging infectious diseases associated with stigmatized populations first by ignoring the disease and later by adopting ineffective and discriminatory public health strategies to try to control it [1-3]. An example of such an approach is the tendency of governments to blame "foreigners" for the introduction and spread of disease, and to place isolation, quarantine, or entry restrictions on this group without regard to actual public health impact [4-8].

In response to the HIV/AIDS epidemic, countries have adopted a wide range of laws and policies that are contrary to effective public health and that violate human rights standards against discrimination, including laws that criminalize HIV transmission [6,9,10], isolate people living with HIV (PLHIV) [6], and censor factual information about safer sex and drug use [11-19]. Laws and policies have also been adopted specifically targeting men who have sex with men [20,21] and migrants [22] because they are perceived to have high rates of infection, including bans against blood donations [20,23]. In the past 20 years, some of these policies have been reversed. But many countries still impose restrictions on entry, stay and residence that prevent PLHIV from legally entering, transiting through, or residing in a country solely based upon their HIV status.

Although international human rights law allows for the restriction of rights in the face of public health emergencies, such restrictions must be the minimum intrusion necessary to effectively address the public health concern. This concept has been articulated in the Siracusa Principles on the Limitation and Derogation Provisions in the International Covenant on Civil and Political Rights, which reflect the broad understanding of human rights as a balance between the rights of individuals and the interests of the community [24]. HIV-related restrictions on entry, stay and residence, however, can be considered both overly intrusive and ineffective public health policy. This article will outline the negative societal and individual health effects, as well as the discriminatory human rights implications of such restrictions, and will call for repeal and reform of these laws and policies.

## Discussion

Although governments have committed in the 2001 Declaration of Commitment on HIV/AIDS, and in subsequent declarations, to enact appropriate legislation to eliminate all forms of discrimination against PLHIV [25], as of September 2008, 66 of the 186 countries in the world for which data were available placed special entry, stay, or residence restrictions on PLHIV [26].

These restrictions take two general forms. The first is an absolute ban on entry for PLHIV, and the second involves restrictions on longer term (generally greater than three months) residence. While no single definitive source has addressed the existence of these laws, the most comprehensive database to track them has found that among countries for which information is available, 14 countries either categorically refuse entry of PLHIV or require disclosure (likely leading to exclusion) [26].

The remainder of countries that impose restrictions do not require documentation of HIV sero-status for short-term stays for business, personal reasons, or tourism, but require it for longer stays. In such cases, an HIV-positive result for an individual applying for a long-term student or work permit in a country usually will lead to refusal of entry or deportation [26,27]. These countries often require periodic mandatory HIV testing of resident non-nationals, and deport individuals who become HIV-positive while residing in the country.

### Public health effects

Migration, defined by the World Health Organization (WHO) as the movement of people from one area to another for varying periods of time [28], is a major global issue with important public health repercussions. The International Organization for Migration has estimated that 192 million people worldwide live outside of their place of birth [29], and the United Nations World Tourism Organization estimates that there were 900 million international tourist arrivals in 2007 [30]. According to WHO, individuals who travel or migrate face serious health risks due to "discrimination, language and cultural barriers, legal status and other economic and social difficulties" [31]. The international community has long been aware of a connection between migration and the risk of HIV infection, though this awareness has not always translated to improved access to HIV-related services [32].

WHO first concluded in 1987 that screening international travelers was not an effective strategy to prevent the spread of HIV [33] and advised in 1988 that such screening would be impractical and wasteful [33]. The Office of the United Nations High Commissioner for Human Rights and the Joint United Nations Programme on HIV/AIDS (UNAIDS) have unequivocally stated that "any restrictions on these rights [to liberty of movement and choice of residence] based on suspected or real HIV status alone ... cannot be justified by public health concerns" [34] since while HIV is infectious, it cannot be transmitted through casual contact [27,35].

HIV-related restrictions on entry, stay and residence may, in fact, negatively impact public health for several reasons. First, these restrictions contribute to and reinforce stigma and discrimination against migrant PLHIV [36] by lending credence to the idea that non-nationals are a danger from which the national population must be protected [22], and by prejudicially implying that PLHIV will act irresponsibly in transmitting the infection [37]. The restrictions make it difficult to discuss and address HIV issues in public, decreasing prevention, testing, and treatment opportunities and uptake [27], and further isolating and marginalizing PLHIV [22]. Singling out HIV for entry restrictions and mandatory testing has also been criticized by experts on the grounds that it creates a false sense of security in a country's nationals that only migrants are at risk for HIV [22,38], and that border control rather than other means of prevention will curb the spread of HIV/AIDS [39].

### Individual impact

The effects that long-term entry, stay, and residence restrictions have on individuals – including students, asylum candidates, and labor migrants – can be devastating. UNAIDS has determined that the greatest impact of HIV entry, stay, and residence restrictions lies on labor migrants [35], of whom there are approximately 86 million worldwide [40]. Frequently, unskilled or semi-skilled labor migrants are subject to mandatory HIV testing prior to departure, are unable to work overseas if found to be positive, and are subject to regular mandatory testing during residence overseas, with summary deportation resulting from a positive test [35]. While few studies have as yet addressed the impact of these restrictions, a 2007 study on the effects of mandatory HIV testing found that migrants' human rights are disregarded throughout the pre- and post-migration process, especially in the lack of informed consent to HIV testing, meaningful HIV test counseling, confidentiality of test results, referral for treatment and support, and in the prospect of immediate deportation from the migrant's destination country [41]. A 2005 study found that HIV-positive Filipino migrant workers in numerous destination countries were deported without counselling or ability to claim unpaid wages or possessions, and were, in some cases, confined in a hospital pending deportation (in one case in Saudi Arabia for as long as 11 months) [42]. Human Rights Watch has also documented pre-departure HIV testing without informed consent, confidentiality or access to test results of prospective migrant workers in Sri Lanka [43] and the deportation of migrants who test positive for HIV from Saudi Arabia [44]. These human rights violations are exacerbated by the fact that they take place with little or no effort to extend HIV prevention, treatment, support, or counseling geared specifically toward this population in either the home or destination countries [35]. Indeed, human rights groups have reported the poor quality of HIV/AIDS care available to detained U.S. immigrants in government-run facilities pending deportation [45], and anecdotal reports have confirmed cases of individuals facing death in deportation confinement without any access to health care [26].

Additionally, HIV-related restrictions on long-term stay and residence can have extremely significant effects on individuals seeking asylum. The fear of HIV testing and the immigration consequences of a positive test result can serve to deter asylum candidates from using legal immigration channels, just as it can for labor migrants [46], therefore increasing the potential for high risk behavior, especially given undocumented immigrants' difficulty finding lawful employment [46]. The United States systematically denies entry to HIV-positive asylum seekers located outside the country [46], unless the individual obtains a waiver [47]. To highlight the consequences of this policy, in 1991, the United States denied entry to 115 HIV-positive Haitian political refugees and their families who otherwise would have been eligible for refugee status under the general criterion used for Haitian asylum seekers at that time. These individuals were detained, along with their family members, at Guantánamo Bay under harsh conditions for over 18 months [46]. UNAIDS has also noted the potentially harmful consequences for refugees when an entire family is migrating and must decide whether to forego migration to a country entirely or to leave an HIV-positive family member behind [27]. As for other detainees, detention of HIV-positive asylum seekers in removal facilities can have severe short- and long-term health consequences [22].

While restrictions on longer-term stays can been detrimental to HIV-positive labor migrants, asylum candidates, and others, the impact of restrictions on short-term entry, stay, or residence can also be serious for affected individuals, including tourists, individuals seeking to visit family, and business travelers. When PLHIV are confronted with questions calling for HIV status disclosure on visa application forms, they must choose between either not disclosing their HIV status (potentially committing fraud and, if caught, risking future entry), and having to hide medication, or disclose their HIV status, and face refusal of entry [26]. Additionally, when entering the United States (which effectively bans HIV-positive travellers from entry absent a waiver visa), those who disclose HIV-positive status and obtain a waiver visa for travel are left with an indelible stamp in their passports, which is visible to travelling companions and to border officials around the world. Confronted with this dilemma, a 2006 study of HIV-positive travellers from the United Kingdom to the U.S. found that of the 239 patients taking antiretroviral therapy (ART) at the time of travel to the U.S., the majority travelled illegally without a waiver visa. Twenty-seven (11.3%) stopped ART during the period of travel, thus running the risk of developing drug resistance. Twenty-eight patients (11.7%) mailed their medication to the U.S. in advance, but only 25% received mailed medication on time [47]. Overwhelmingly, individuals who stopped treatment reported doing so because of the U.S. travel restrictions, fear of being searched, and discovery of their illness [48]. Additional studies on the behaviour of HIV-positive international travellers have also found high rates of non-compliance with ART during travel [49].

### Human rights implications

National restrictions on entry, stay, and residence for PLHIV broadly violate international human rights law provisions banning discrimination and upholding equality before the law [50-54]. Following the Universal Declaration of Human Rights [50], the International Covenant on Civil and Political Rights (ICCPR) guarantees all persons the right to equal protection of the law without discrimination based on race, color, sex, language, religion, political or other opinion, national or social origin, property, birth or other status [51]. The U.N. Commission on Human Rights has interpreted this provision to include discrimination based on HIV status [55]. States must respect this right for all individuals within their territory and subject to their jurisdiction [56], regardless of citizenship [57]. Indeed, the Human Rights Committee – the ICCPR's monitoring body – has noted that " [i]t is in principle a matter for the State to decide who it will admit to its territory. However, in certain circumstances an alien may enjoy the protection of the Covenant even in relation to entry or residence, for example, when considerations of non-discrimination, prohibition of inhuman treatment and respect for family life arise" [57].

Human rights bodies, such as the European Court of Human Rights, have concluded that states have little freedom to implement entry and residence policies and laws that clearly discriminate against particular groups [58,59]. The Convention on the Rights of the Child also requires that the rights it guarantees be respected without discrimination [52], a provision that has been interpreted to include discrimination based on HIV status [60]. Thus, bans on the travel or immigration of HIV-positive children – for example in the case of international adoption – would be specifically prohibited.

Restrictions against entry, stay, and residence based on HIV status also run contrary to related human rights principles. As UNAIDS has noted, the implementation of these restrictions has regularly violated the human rights principle of *non-refoulement *of refugees (which prohibits return to a place where life or freedom is threatened) [34], obligations to protect the family, protection of the best interests of the child, the right to privacy, the right to freedom of association, the right to information, and the rights of migrant workers [27]. These restrictions also affect the individual's rights to seek asylum and to work, as well as the rights to education, the highest attainable standard of health, dignity, and life.

According to international human rights law, as noted above, to avoid being classified as impermissible discrimination, any difference in treatment that has a negative impact on a particular group – e.g. persons living with HIV or AIDS – has to be justified by being necessary to achieve a compelling purpose and be the least restrictive (meaning least discriminatory) means of achieving that purpose [24,27]. However, while preservation of public health is a compelling purpose that might justify some forms of restrictions, HIV-related distinctions in entry, stay, and residence do not actually protect public health, and are too broad and coercive [34] to be the least restrictive means to achieve this end [27,61].

### Looking Forward

An increasing awareness of the discriminatory nature and deleterious effects of HIV-related travel laws has begun to prompt change. In 2004, El Salvador made the decision to remove HIV-related entry, stay, and residence regulations [26]. In advance of the International AIDS Conference in Toronto in 2006, Canada eliminated requirements of disclosure of HIV status for short-term stays. China indicated in 2007 that it intends to remove all of its restrictions on PLHIV entering the country [62,63], and the United States also has made a commitment to eliminate restrictions [64], though neither country has yet fully done so. Numerous organizations, states, and individuals have rallied, asking countries to eliminate HIV-related entry conditions [26], and prominent bodies such as the International AIDS Society have refused to hold conferences in countries that persist in these restrictions. However, as some countries have been relaxing their restrictions, others have moved in the direction of tightening [26,47,65,66].

Human rights and HIV/AIDS organizations must continue to demand that such restrictions be repealed immediately and entirely. In instances in which these laws and policies are not rescinded, at a minimum national governments need to reform testing practices so as to conform with basic human rights standards. Conducting voluntary testing, obtaining informed consent, and providing adequate pre- and post-test counseling are key to ensuring that the rights of involved individuals receive a minimum measure of respect [27,47]. Confidentiality of test results should also be strictly maintained. Policies subjecting individuals to expulsion must always be coupled with protection of that individual's right to challenge his or her deportation through due process of law [27]. As UNAIDS has demanded, " [r]estrictions against entry or stay that are based on health conditions, including HIV/AIDS, should be implemented in such a way that human rights obligations are met, including the principle of non-discrimination, *non-refoulement *of refugees, the right to privacy, protection of the family, protection of the rights of migrants, and protection of the best interests of the child. Compelling humanitarian needs should also be given due weight" [27].

Regardless of a country's policies on HIV-related travel restrictions, provision of adequate HIV/AIDS prevention, care, and treatment services for migrants and citizens alike is essential [35]. As noted above, the experience of discrimination, dislocation and disruption in social networks around migration is closely linked to HIV risk. Legislative priority and government resources should be redirected from maintaining costly and discriminatory entry, stay, and residence restrictions on PLHIV, and refocused on providing prevention, care, and treatment programmes that target and serve non-citizens and citizens. The creation and maintenance of such programmes will be the truly effective long-term strategy in combating this pandemic from both a public health and a human rights perspective.

More than twenty-five years since HIV was first identified, laws and policies such as entry, stay and residence restrictions for PLHIV, based solely upon unfounded fear and ignorance, should be eliminated.

## Summary

Although governments have committed in the 2001 Declaration of Commitment on HIV/AIDS to enact appropriate legislation to eliminate all forms of discrimination against persons living with HIV (PLHIV), as of August 2008, 67 of the 184 countries in the world for which data were available placed special entry, stay, or residence restrictions on PLHIV. These discriminatory restrictions are not justified by public health rationales and indeed have been criticized for their negative effect on public health, both on society as a whole and on individuals, including labor migrants, asylum candidates, and short-term travelers. These restrictions need to be eliminated immediately and national governments need to refocus their legislative efforts and resources devoted to HIV/AIDS on effective prevention, care, and treatment programmes serving citizens and non-citizens that accord with human rights law.

## Competing interests

The authors declare that they have no competing interests. This research was supported by Human Rights Watch, an independent, nongovernmental organization.

## Authors' contributions

JJA conceived the manuscript. JJA and KWT reviewed the literature and wrote the manuscript. Both authors read and approved the final manuscript.
